# Bone Health and Its Relationship with Impact Loading and the Continuity of Physical Activity throughout School Periods

**DOI:** 10.3390/ijerph16162834

**Published:** 2019-08-08

**Authors:** Gotzone Hervás, Fatima Ruiz-Litago, Jon Irazusta, Amaia Irazusta, Begoña Sanz, Javier Gil-Goikouria, Ana Belen Fraile-Bermudez, Carmen Pérez-Rodrigo, Idoia Zarrazquin

**Affiliations:** 1 Department of Physigology, Faculty of Medicine and Nursing, University of the Basque Country (UPV/EHU), 48940 Leioa, Spain; 2 Department of Nursing I, Faculty of Medicine and Nursing, University of the Basque Country (UPV/EHU), 48940 Leioa, Spain

**Keywords:** bone health, growth and development, children, adolescents, exercise, lifestyle change, muscle–skeletal system

## Abstract

Bone is influenced by physical activity (PA) throughout life, but childhood and adolescence provide a key opportunity to maximize peak bone mass. Thus, it is important to identify the relationship between PA practiced in childhood and young adulthood to design a promotion plan for bone health. The purpose of this study was to analyze the relationship between different impact-loading PAs (and their continuity throughout school periods from childhood to young adulthood) and bone stiffness index (SI). In this cross-sectional study, which was conducted on 145 university students aged 18–21 years, bone measurements were measured by quantitative ultrasonometry (QUS), and PA information was recalled using a self-administered questionnaire. Associations between the SI and the impact of PA performed during secondary school (*p* = 0.027), high school (*p* = 0.002), and university (*p* = 0.016) periods were observed. The continuity of PA over a longer period of time was related to a higher SI (*p* = 0.007). Those who practiced PA throughout all school periods had a higher SI than those who practiced during primary school only (*p* = 0.038) or through primary and secondary schools (*p* = 0.009). These results suggest that impact-loading PA practiced during different school periods is related to higher values of the SI. Therefore, continuous PA from an early age may be an important contributing factor to achieving and maintaining adequate bone health.

## 1. Introduction

Osteoporosis is a serious threat to public health. With progressive aging of the population, the number of people affected by this bone-weakening disease is rapidly growing. In Europe, it is estimated that the incidence of osteoporotic fractures will increase by 28% by 2025 [1]. Although disorders associated with decreased bone health manifest in adulthood [2], prevention begins at an early age, when peak bone mass (PBM) has not yet been reached [3,4]. During childhood and adolescence, bone deposition predominates over resorption, so these periods are critical for the adequate development of PBM. Further, higher PBM in young adulthood is associated with reduced bone fragility later in life [5]. Therefore, the childhood and adolescent years provide an optimal period to maximize bone mass and strength.

Bone mineral content and structure, besides being affected by genetics, hormones, and nutrition, are influenced by physical activity (PA) [6,7]. Impact loading, which is inherent in many sports, plays an important role in skeletal growth and shaping [8]. Previous studies of children and young adults have shown that impact-loading PA is associated with a greater bone mineral density than nonimpact activities [9,10].

Most studies analyzing the relation between bone characteristics and impact-loading PA have examined young athletes with unique characteristics, such as a higher level of training than the general population [11,12]. However, few studies have analyzed bone status and impact-loading exercises in a young general population. Children and young adults practice a wide variety of PA: it therefore might be important to identify which activity types are most beneficial to improve bone health during each growth period and in the long term.

Some studies have demonstrated the bone-related benefits of exercise at an early age [13,14], confirming the osteogenic effects of PA throughout childhood and adolescence. These effects are long-lasting: even a short-term and high-impact PA intervention in early childhood exerted effects up to seven years after the program ended [15]. In addition, reduced sports activity during young adulthood has been related to decreased bone mineral content [16]. However, as far as we know, few studies have analyzed the relation between PA performed in previous periods of life and bone status in young adulthood [17].

Appropriate activities and their continuity of practice must be identified before developing prevention strategies that will have the greatest public health impact on bone health. The different types of sports practiced during school periods are regulated activities [18], and the standardized characteristics of each allows us to associate its practice with current health parameters and to define its impact on them. Therefore, the objective of this study was to identify which types of impact-loading PAs are related to enhanced bone stiffness throughout childhood and young adulthood and the relevance of PA continuity.

## 2. Methods

### 2.1. Participants

This was a cross-sectional study: 156 young adults consented to participate in this study. Results were obtained from 145 of them (56 men and 89 women), aged 18–21 years (18.72 ± 0.76 years). The study was carried out from September 2016–May 2017. All participants were recruited from different university degree programs at the University of the Basque Country (Leioa, Spain). The Research Ethical Committee of the University of the Basque Country approved the study (M10/2015/122), and it was conducted in accordance with the Declaration of Helsinki. In accordance with ethical standards for human experimentation, all participants received verbal and written information about the purpose and procedure of the study and provided voluntarily signed informed consent before starting the investigation. The inclusion criteria were being between 18 and 21 years and not having any current bone injury, metabolic alterations during puberty, hormonal dysfunctions in the growing period, previous dietary problems, or other health problems that could alter bone metabolism (hormones, vitamin D, etc.).

### 2.2. Bone Status

Bone status was measured on the right calcaneus using Lunar Achilles Insight (GE Healthcare, Milwaukee, WI, USA). This noninvasive, radiation-free quantitative ultrasonometry (QUS) method was developed to assess bone mineral status in peripheral skeletal sites. Correlations between the stiffness index (SI), the bone mineral density, and content measured by dual energy X-ray absorptiometry have been reported in children and adolescents [19]. Thus, the SI is a suitable QUS parameter to analyze bone health. The SI is calculated by the raw parameters of broadband ultrasound attenuation and the speed of sound and indicates bone trabecular complexity, architecture, and elasticity. Better bone structure absorbs more sound waves, resulting in higher SI values.

The SI value indicates bone stiffness, so a higher SI is related to better bone health. We used *Z*-scores based on the SI as a basis for classifying bone health into three groups: normal (*Z*-score > −1), osteopenia (*Z*-score = −1–−2.5), and osteoporosis (*Z*-score <−2.5).

Before each screening session, the bone densitometer was calibrated according to the manufacturer’s standard procedures, and all measurements were performed per the manufacturer’s recommendations.

### 2.3. Impact-Loading PA

Participants completed a self-administered questionnaire about their PA, excluding that performed in physical education classes, during different school periods: primary school (6–11 years), secondary school (12–15 years), high school (16–17 years), and university (18–21 years). Participants also provided information on the type of PA practiced, with a minimum dedication of 2 sessions/week. It was also considered if the PA was federated or not. Federated sports players in Spain are defined as those who are officially recognized for their sport by a sanctioned sports association. They are regulated by their respective federations and have formal competition. Twenty-nine different types of PA were reported. The different PAs were classified into 5 types of loading (Table 1) in accordance with classifications used in similar studies [11], with no PA as a 6th category.

### 2.4. Continuity of PA

To analyze the importance of activity consistency, we assessed PA across different periods of schooling. Except for PA performed in physical education classes, participants indicated on a self-administered questionnaire whether they had practiced any PA during different school periods. Participants were categorized into 5 groups according to the time they had practiced PA independently of the type of impact: only during primary school, through secondary school, through high school, continuously from childhood to university periods, and noncontinuously throughout school periods.

### 2.5. Statistical Analyses

Data were analyzed using SPSS software version 24.0 (IBM Corp., Armonk, NY, USA). Bone descriptive statistics are presented as mean and SD. The frequency of impact loading and federated participants in each type as well as the continuity of PA in different school periods were calculated. Differences in the bone SI between different impact-loading modalities and the tracking of physical activity throughout the participants’ lives were estimated by analysis of covariance (ANCOVA) using sex as a covariate. When differences were statistically significant, the least significant difference (LSD) post hoc test was set to compare the means of two categories, and *p* < 0.05 was considered statistically significant.

## 3. Results

### 3.1. Descriptive Characteristics of the Study Population

Here, 145 young adults (56 men and 89 women) who had an average age of 18.72 ± 0.76 years participated. Participants’ bone SI (117 ± 20) and bone quality, measured by a *Z*-score (1.44 ± 1.5), showed values within the references of normality. There were no observed significant differences between male and female values.

Data for the sample distribution in relation to different impact-loading PA throughout school periods are displayed in Table 2. An analysis of PA modalities according to impact loading showed that odd-impact loading was most prevalent in all school periods. However, the number of participants who practiced this type of PA gradually decreased with progressing school periods, from 55.9% of participants in primary school to 21.4% in university period. High-impact PA also had high participation at an early age, but similarly decreased with progressive school periods. This was contrary to repetitive low-impact and high-magnitude PA, which were practiced most from high school onwards.

Federated activities accounted for 80.9% of PA practiced in secondary school and 67.5% of PA in high school. These percentages were significantly higher than those estimated for primary school (33.3%) and the university period (49.5%). In all school periods, high-, odd-, and nonimpact-loading PAs were mostly federated. However, low-impact and high-magnitude PAs were mainly nonfederated.

The different continuity of PA throughout school periods is shown in Table 3. The number of individuals who did not practice PA increased considerably with progressing school periods. Across school periods, 58.6% of participants continuously practiced PA from primary school to university. In primary school, 91.7% of participants practiced PA throughout different school periods: once they arrived at university, 33.1% stopped practicing PA without resuming the practice in later periods. This stop occurred most frequently during high school. Even so, 7.6% of participants who stopped practicing PA did resume the activity in a later period.

### 3.2. Impact-Loading PA Was Associated with Bone Status

To determine the relationship between different impact-loading PAs and the bone SI, we performed an analysis of covariance. As shown in Table 4, the bone SI was significantly associated with PA performed during secondary school (*p* = 0.027), high school *(p* = 0.002), and university (*p* = 0.016) periods. Not practicing any PA at secondary school was related to a lower bone status than high-impact PA (*p* = 0.033) was. High- and odd-impact PAs were also related to a better bone SI than not practicing any PA during high school (*p* = 0.001, *p* = 0.002) and university periods (*p* = 0.006, *p* = 0.012) was. Among impact exercises, high-impact PAs practiced during primary school were related to a higher bone SI than nonimpact PAs (*p* = 0.028) were. Similarly, those who practiced high-impact PAs had a better SI than those who practiced repetitive low-impact PAs during secondary school (*p* = 0.038) and university periods (*p* = 0.040).

### 3.3. PA Continuity Was Associated with Bone Status

The association between the SI and the continuity of PA practice throughout school periods is reported in Table 5 through an analysis of covariance. The practice of PA over a longer period of time was significantly related to better bone health (*p* = 0.007). Participants who had continuously practiced PA throughout all school periods had a higher bone SI than those who only practiced during primary school (*p* = 0.038) and through primary and secondary school (*p* = 0.009).

## 4. Discussion

We analyzed bone status in a group of university students without metabolic alterations or any dysfunctions that could alter bone metabolism according to PA practiced from childhood through university. As we might have expected, our data suggested that individuals who practiced high- and odd-impact PAs had enhanced bone SI values compared to those who practiced low-impact activities or did not regularly practice PA. Likewise, the practice of PA without interruption from primary school to university periods was related to a higher bone SI. These results agree with previous work that described the positive effects of the impact of PA on the bone SI [8].

The analysis of PA during different school periods showed that those who practiced high- or odd-impact-loading PA had higher SI values. Similar studies have shown that high-impact loading may be the most effective impact-loading PA to optimize bone during adolescence [20,21]. In addition, participation in ball sports, which were included as high- and odd-impact PAs, may represent a strategy to promote bone health in young people. One potential explanation is that multidirectional loading and high ground reaction forces in ball sports may result in stiffer and more fracture-resistant bones [22].

Few studies have analyzed the association between the bone SI and different types of impact-loading PA during an extended period in a young population [23]. In the present study, we did not observe significant differences in the bone SI according to the type of PA practiced during primary school (*p* = 0.082). However, from secondary school onwards, different types of impact-loading PAs were significantly associated with the bone SI. This could have been because for primary school children, PA is influenced by their activities during school recess periods and physical education classes. Therefore, overall PA may be more homogeneous for primary school children, meaning the responses generated through different types of impacts might be similar. Moreover, federated PA becomes more frequent from secondary school onward. Federated PA is competitive, so these activities might be more organized and structured than nonfederated activities, contributing to increased differences between activities.

Due to changes in PA habits and behaviors over time, the effects of impact loading on the bone SI were more evident in secondary school, high school, and university periods. However, bone appears to be most responsive to mechanical stress from midpuberty to menarche or during Tanner stages II–IV [24]. Age-related changes in hormonal status and body composition related to the maturity of the participants in different school periods therefore may have influenced the response of the bone SI to different impact-loading PAs.

High-impact PA in primary, high school, and university periods was related to the highest bone SI values. However, the highest SI values in secondary school individuals were detected in those who performed nonimpact PAs. The muscle component plays an important role in these types of activities, which include swimming, water polo, canoeing, spinning, and surfing. There is a strong link between bone and muscle development in children, suggesting that increasing muscle mass during growth stimulates bone accrual [25,26,27]. Thus, PAs that promote bone growth could include not only high-impact PAs, but also nonimpact PAs, because they increase muscle mass and strength, creating tension in skeletal structures and thus indirectly contributing to bone health [28]. In addition, the bone SI of participants who performed nonimpact-loading PAs from high school onwards did not significantly differ from those who practiced high-magnitude loading. Bearing in mind the dynamic component and the muscle demands of high-magnitude and nonimpact-loading activities, they might provide adequate stimuli to generate osteogenic stress and strain reactions in bones. Moreover, cellular interactions between both tissues have been investigated. Recent studies have described a bidirectional signaling cytokine system between bone and muscle tissues during all stages of osteocytes and myoblast differentiation [29], showing that muscle-derived myokines exert a paracrine function, providing a bone anabolic effect [30,31]. Thus, an increased muscle mass would lead to a higher secretion of myokines, which could have positive effects on bone density.

Systematic reviews and meta-analyses have reported a negative relation between nonimpact sports and bone [32] and have even equated them to values for a sedentary population [33]. Nevertheless, in accordance with our results, a meta-analysis of the influence of swimming on the BMD during childhood and adolescence concluded that, in bone, small differences emerge between swimmers and high-impact athletes at the beginning of adolescence, but that those differences appear to increase over time because of cumulative effects [34].

Activities classified as repetitive low-impact loading, such as zumba, aerobics, dance, or jogging, are exercises that generally are nonfederated because they are often performed at lower intensities. A study examining the potential effects of early childhood moderate and vigorous physical activity (MVPA) on later bone health found that increased MVPA during childhood contributed to an increased BMC and sustained bone health [35]. However, another study also concluded that the same duration of more intense or vigorous PA had a greater association with the bone SI than MVPA did [36]. In addition to PA impact type, the fact that repetitive low-impact-loading PAs can be practiced at lower intensities also could account for their lower association with the bone SI than other impact-loading PAs.

With regard to PA continuity, pre- and peripubertal periods are considered sensitive periods for bone stimuli [37]. Puberty is a critical time for bone development, as 26% of final bone mass is attained between ages 11.5 and 13.5 years in girls and 13 and 15 years in boys [38]. Improvements in bone status during those periods have been associated with a higher PBM and a decreased risk of osteoporosis later in life [39]. Thus, due to its osteogenic capacity, PA during adolescence greatly influences the bone SI and may delay the onset of osteoporosis [40].

Our data showed that the number of individuals who did not practice PA beyond 2 h of physical education classes increased with progressing school period. The most inactive participants, those who did not practice any PA apart from that performed in physical education classes, had the lowest bone SI in all school periods analyzed. Nonphysically active youth have been shown to have a lower PBM compared to youth undergoing regular PA during the growth phase [41]. Further, physical inactivity has deleterious effects on bone health [42]. Mechanistically, not practicing PA may negatively influence bone turnover and the achievement of proper PBM by disrupting the bone formation–resorption balance [43]. On the basis of skeletal homeostasis, the osteocyte, which acts as a bone mechanosensor and as an orchestrator of osteoblast and osteoclast activity, modulates bone remodeling depending on the type of load perceived [44].

The results of this study also suggest that a high bone SI necessitates ongoing PA over time. Previous work has shown that former athletes who do not maintain participation in sports may retain some bone benefits, but their BMD is lower than athletes who continue to participate in sports [8]. Another study has shown that jumping activities during early childhood increase bone mass and that this gain is maintained even after discontinuing the activity [45].

Intervention studies have shown that 10-week, 3-month, and 20-month PA programs may be enough to produce changes in the bones of school-aged children [46,47,48]. However, it is important to note that these changes must be maintained over time to be effective in the long term. Continued PA promotes skeletal homeostasis, and bone shape, mass, and structure adaptations are due to current mechanical needs of skeletal segments [49].

The duration of the intervention’s effect in these studies was not analyzed, although our results suggest the importance of continuous and sustained exercise throughout all school periods, from prepubertal to young adulthood, to enhance the bone SI.

There were some limitations to this research that should be considered. Due to the cross-sectional nature of this study, causality cannot be inferred from our results. In addition, bone stiffness does not analyze bone mass, density, and geometry separately, giving only an estimation of overall bone mineral status. Nonetheless, this limitation did not preclude reliable results. The simplicity, versatility, low cost, and lack of ionizing radiation of QUS have helped disseminate this method worldwide. Furthermore, the reporting of PA from years prior to the point of data collection was reliant upon self-reported information, which could have introduced bias to the data. However, the occasional practice of physical activity was not considered. PA should be practiced at least with a minimum dedication of two sessions per week over 8–10 months.

A strength of this study was its novel examination of the association between bone stiffness and PA throughout school periods from an impact-loading perspective. We are not aware of any studies in which these variables that could affect bone stiffness were analyzed in a young general population. To date, most studies have analyzed the association between impact loading and bone in athletes, and thus they were not comparable to a general population.

## 5. Conclusions

Promoting healthy habits throughout life increases the prospects for a healthier life. PA is one of the main factors related to the promotion of community health. The PA carried out in school sports is based on standards. Therefore, the PA done through the sports practiced during different school periods, classified according to their type of impact, contribute to the organization and implementation of public health policies. In that way, the results of this study can contribute to the design of health promotion and intervention plans for improved bone health from an early age. It is also important that health professionals, who provide health guidelines to patients and the general population, understand the basis for how and in what way, across stages of childhood and adolescence, PA habits promote bone health.

The results indicated that impact-loading PA had a positive relationship with bone SI. This may have been due to impact loading-specific differences, a bone–muscle connection, or because the practice of PA was related to a more active lifestyle. In school policies, the promotion of impact-loading activities participation might be an effective strategy to improve bone health in adulthood. Similarly, maintaining PA throughout school periods, from childhood and adolescence to young adulthood, is associated with better bone health. Therefore, continued PA, as well as promoting a healthy lifestyle at an early age, may be an important contributing factor to achieve and maintain high bone SI. This factor should be considered not only at early life periods before reaching PBM, but also at later periods when interventions to prevent bone loss focus on maintaining bone density and stiffness.

## Figures and Tables

**Table 1:** Physical activity (PA) characteristics and sports included in each category.

Impact-Loading	Characteristics	Sports
High-impact	Includes maximal vertical jumps and ground impacts	Volleyball
		Basketball
		Handball
		Ballet
		Rhythmic gymnastics
Odd-impact	Involves rapid turns and stops with ground impacts	Football
		Tennis
		Field hockey
		Multisport *
		Karate
		Judo
		Boxing
		Rugby
		Basque pelote
		Ice skating
		Crossfit
High-magnitude	Slow and well-coordinated movements that apply maximal muscle force without ground impacts	Weightlifting
Repetitive low-impact	Ground impacts during long-lasting performances at a relatively constant speed	Jogging
		Aerobics
		Zumba
		Modern dance
		Tug of war
		Ping pong
Repetitive nonimpact	Muscle forces during long-lasting performances without ground impact	Water polo
		Swimming
		Spinning
		Surfing
		Canoeing
		Equestrianism

* Multisport: An after-school sport activity in which different sports such as athletics, football, and basketball were practiced as an initiation and a step, before practicing a specific sport in which physical and sport-specific skills were strengthened.

**Table 2:** Participant allocation by impact-loading PA in each school period.

Impact-Loading	Primary School (6–11 Years)		Secondary School (12–15 Years)		High School (16–17 Years)		University (18–21 Years)	
	*n* (%)	Federated *	*n* (%)	Federated *	*n* (%)	Federated *	*n* (%)	Federated *
		46 (33.3)		110 (80.9)		79 (67.5)		46 (49.5)
High-impact	32 (22.1)	11 (34.4)	41 (28.3)	40 (97.6)	31 (21.4)	30 (96.8)	20 (13.8)	15 (75)
Odd-impact	81 (55.9)	30 (37)	64 (44.1)	54 (84.4)	44 (30.3)	37 (84.1)	31 (21.4)	21 (67.7)
High-magnitude	–	–	–	–	3 (2.1)	0	8 (5.5)	0
Repetitive low-impact	7 (4.8)	1 (14.3)	16 (11.1)	4 (25)	20 (13.8)	3 (15)	24 (16.5)	3 (12.5)
Repetitive nonimpact	18 (12.4)	4 (22.2)	15 (10.3)	12 (80)	19 (13.1)	9 (47.4)	10 (6.9)	7 (70)
Nonsport	7 (4.8)	–	9 (6.2)	–	28 (19.3)	–	52 (35.9)	–

* Federated: Those who have official recognition for their sport by a sanctioned sports association. They are regulated by their respective federations and have formal competition.

**Table 3:** Participant allocation by continuity of PA in each school period.

Continuity	*n* (%)
Nonsport	1 (0.7)
Continuous	
* Primary school* (6–11 years)	133 (91.7)
* Secondary school* (12–15 years)	127 (87.6)
* High school* (16–17 years)	114 (78.6)
* University* (18–21 years)	85 (58.6)
Noncontinuous	11 (7.6)

**Table 4:** The bone SI by impact-loading PA during different school periods, adjusted for gender.

Impact-Loading	Primary School (6–11 Years)		Secondary School (12–15 Years)		High School (16–17 Years)		University (18–21 Years)	
	Mean ± SD	*p*	Mean ± SD	*p*	Mean ± SD	*p*	Mean ± SD	*p*
		*0.082*		*0.027*		*0.002*		*0.016*
High-impact	123 ± 18 #		120 ± 19 †,‡		124 ± 18 ‡		127 ± 17 †,‡	
Odd-impact	117 ± 20		119 ± 22		122 ± 21 ß		124 ± 24 ß	
High-magnitude	—		—		116 ± 33		118 ± 21	
Repetitive low-impact	121 ± 20		107 ± 11		114 ± 8.7		114 ± 13	
Repetitive nonimpact	110 ± 20		123 ± 17 §		117 ± 22		117 ± 25	
Nonsport	105 ± 20		102 ± 19		104 ± 18		111 ± 18	

# High-impact versus repetitive nonimpact (*p* < 0.05), † high-impact versus repetitive low-impact (*p* < 0.05), ‡ high-impact versus nonsport (*p* < 0.05), § repetitive nonimpact versus nonsport (*p* < 0.05), ß odd-impact versus nonsport *(p* < 0.05).

**Table 5:** The bone SI by continuity of PA during different school periods, adjusted for gender.

Continuity	Mean ± SD	*P*
		*0.007*
Primary school (6–11 years)	104 ± 22 #	
Secondary school (12–15 years)	104 ± 17 †	
High school (16–17 years)	117 ± 17	
University (18–21 years)	122 ± 20	
Noncontinuous	109 ± 20	

# Primary school versus university (*p* < 0.05), † secondary school versus university (*p* < 0.05).
